# Effect of filler particles on surface roughness of experimental composite series

**DOI:** 10.1590/S1678-77572010000100011

**Published:** 2010

**Authors:** Hanadi Yousif MARGHALANI

**Affiliations:** 1 BDS, MSc, PhD, Assistant Professor, Operative Dentistry Division, Conservative Dental Science (CDS) Department, Faculty of Dentistry, King Abdulaziz University, Jeddah, Kingdom of Saudi Arabia.

**Keywords:** Resin composites, Surface roughness, Roughness parameters, Filler size and shape

## Abstract

**Objective:**

The purpose of this study was to evaluate the effect of different filler sizes and shapes on the surface roughness of experimental resin-composite series.

**Material and Methods:**

Thirty-three disc-shaped specimens of the series (Spherical-RZD 102, 105, 106, 107, 114 and Irregular-RZD 103, 108, 109, 110, 111, 112) were prepared in a split Teflon mold and irradiated with an halogen light-curing unit (450 mW/cm^2^ for 40 s) at both top and bottom surfaces. The specimens were stored for 3 months in distilled water. The surface roughness values in form of surface finish-vertical parameter (R_a_), maximum roughness depth (R_max_) and horizontal roughness parameter (Sm) were recorded using a contact profilometer. The data were analyzed by one-way ANOVA and the means were compared by Scheffé post-hoc test (α=0.05).

**Results:**

The lowest surface roughness (R_a_) was observed in S-100 (0.079±0.013), while the roughest surface was noted in I-450/ 700/1000 (0.125±0.011) and I-450/1000 (0.124±0.004). The spherical-shape series showed the smoothest surface finish compared to the irregular-shape ones with higher significant difference (p>0.05). The vertical surface roughness parameter (R_a_) values increased as the filler size increased yielding a linear relation (r^2^=0.82). On the contrary, the horizontal parameter (Sm) was not significantly affected by the filler size (r^2^=0.24) as well as the filler shape.

**Conclusions:**

Filler particle’s size and shape have a great effect on the surface roughness parameters of these composite series.

## INTRODUCTION

Surface roughness property of the restoratives has long been recognized as a parameter of high clinical relevance for plaque accumulation, staining susceptibility and wear. Increasing esthetic demands from the patients resulted in a wide use of resin composites in dental practice. The structures of resin matrix, coupling agent and the characteristics of filler particles have a direct impact on the surface smoothness of resin composites^6^. The main intrinsic factor that affects surface smoothness of any composite is filler component. The type of inorganic particles, size of fillers, and extend of filler loading are considered the most important factors. Various experimental composites have been introduced aiming to comparatively evaluate their properties in order to increase their optimum clinical performance^1,2,5^.

Highly esthetic and polished surfaces of resin composites can be obtained by minimizing the filler size. The main concept of creating composites with nanofiller particles is to have superior properties, such as strength, stiffness as well as color and thermal stability, to the conventional ones. Lately, one of the important advances in nanotechnology science is their application to dental resin composites as in Filtek Supreme XT^7,10,11^. Nanofill composites are composed of nanomer or nanocluster, whereas nanohybrids are hybrid resin composites with nanofiller in a prepolymerized filler form^11^. Nanofill composites are claimed to offer ultimate esthetics, excellent wear resistance and strength^10^. Surface characteristics of composites in form of roughness, topography and texture have been considered as important parameters of clinical relevance for wear resistance, plaque retention and discoloration susceptibility. In vitro studies have indicated that nanofill resin composites showed favorable mechanical properties as optical and gloss characteristics, reduced polymerization shrinkage, higher surface quality and superior polish^18,21^.

Several studies have been made to study the effects of dental composite’s microstructure on its properties^1,10^. Filler component in term of size, distribution, geometry and volume fraction have been investigated extensively^1,20^. Fundamental understandings of the factors that affect the superior clinical performance of the resin composites can assist in more refinement of these materials during manufacturing. Therefore, this study is aimed to evaluate the effect of different filler sizes ranged from 100 to 1500 nm and geometry (spherical and irregular) on the surface characteristics of experimental resin composite series. The surface roughness was measured from both vertical and horizontal dimensions to give more details on the surface structure of the composite materials. The null hypotheses stated that; (a) there are no differences between surface roughness values of the experimental composite series, and (b) there is no correlation between both vertical as well as horizontal surface roughness parameters and the increase in filler particle size.

## MATERIAL AND METHODS

Eleven series of experimental resin composites based on different filler particle size formulations (range of 100-1500 nm) and two geometries (spherical and irregular) were investigated (Table 1). These series comprised Bis-GMA, UDMA, TEGDMA resin matrix, 0.33% camphorquinone and barium glass particles of 56.7% filler volume fraction. These particulate dispersed phases were systematically graded in size and treated with a silane coupling agent (methacryloxypropyltrimethoxysilane). The spherical particles were silica and made from solution, while the irregular particles were ground glass melts (Ba-Al-B-silicate glass).

**Table 1:** Experimental composite series formulations

**Resin-composite series (Batch #)**	**Code**	**Filler Particles**				**Matrix**	**Manufacturer**
		**Size (nm)**	**Shape**	**Wt%**	**Vol%**		
RZD 102	S-100	100	Spherical	72.3	56.7	Bis-GMA, UDMA, TEGDMA	Ivoclar Vivadent, Schaan, Liechtenstein
RZD 107	S-250	250	Spherical	72.6	56.7		
RZD 106	S-500	500	Spherical	72.6	56.7		
RZD 105	S-1000	1000	Spherical	72.5	56.7		
RZD 114	S-100/250/1000	100:250:1000 (1:1:2)	Spherical	72.0	56.7		
RZD 103	I-450	450	Irregular	76.4	56.7		
RZD 108	I-700	700	Irregular	76.4	56.7		
RZD 109	I-1000	1000	Irregular	76.4	56.7		
RZD 110	I-1500	1500	Irregular	76.4	56.7		
RZD 111	I-450/1000	450:1000 (1:3)	Irregular	76.4	56.7		
RZD 112	I-450/700/1000	450:700:1000 (1:1:3)	Irregular	76.4	56.7		

Thirty-three disc-shaped specimens (10 mm diameter x 2 mm thick) of the composite series were fabricated at room temperature in a split Teflon mold (n=3). The uncured material was gently packed inside the mold which was covered from both sides with thin transparent Mylar strips (KerrHawe Neos Dent, Bioggio, Switzerland). The unset specimen inside the mold assembly was pressed between two microscope glass slides (76 x 26 x 1 mm Surgipath glass) to extrude the excess of the material resulting in a flat surface.

The composite series were irradiated by a conventional halogen light-curing unit (Optilux 501, Demetron/Kerr, Danbury, CT, USA) at 450 mW/cm^2^ for 40 s at both top and bottom surfaces. The specimens were exposed to the same amount of irradiation after removal of the glass slides and Mylar strips. The power density of the curing-unit was monitored with an external radiometer (Demetron/Kerr, Danbury, CT, USA) before curing the specimens. Then the cured specimens were removed from the mold and stored in dark bottles of 30 ml capacity containing distilled water at 37± 0.5ºC for 24 h to complete polymerization of the material. Afterwards, they were lightly finished manually from the top-surface with 1000-grit silicon carbide (SiC) abrasive paper under running water and polished with 1500 SiC paper as well as 5 and 1 µm aluminum oxide slurry pastes for 5 sec each step. This will allow removal of a weak resin-rich layer yielding a uniform surface finish. The samples were sonically cleaned in distilled water for 15 min, stored for 3 months at 37ºC incubator, and were then blotted dry with an absorbent paper before measurement of roughness parameters.

The examined surface was assessed for any artifacts such as pores or scratches by stereo-microscope (Meiji Techno America, San Jose, CA, USA) because those with defects were discarded and replaced with new ones. The surface roughness parameters were measured by a contact profilometer (Surfcorder SE 1700; Kosaka Corp., Tokyo, Japan) equipped with a 5-µm radius diamond-tipped stylus that was attached to a pickup head. The stylus traversed the surface of the specimen at a constant speed of 0.5 mm/s with a force of 4 mN and automatic return. Each specimen was traced in three parallel locations near the center across the top-surface with an evaluation length of 4 mm. The data were filtered with a cut-off (λc) of 0.8 mm (Gauss profile-Filter) and the tracings were 0.8 mm in length because the standard JIS94 was selected as a measuring profile. Leveling of all parts of the apparatus can be achieved by adjusting the pick-up head knob.

**Figure 1:**
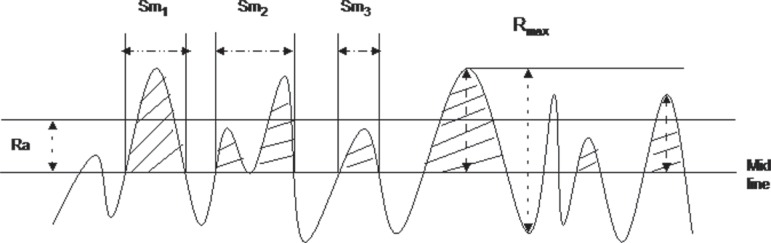
Surface roughness parameters selected, R_a_, R_max_, and Sm

Preparation and finishing of specimens were performed by only one operator. The accurate performance of the profilometer was checked periodically by the use of a calibration block. The surface irregularity signals were transformed into digital values that monitored on a computer. The following roughness parameters were selected to describe the surface texture of the investigated composite series^4,12^. The parameters are illustrated graphically in Figure 1.

R_a_ is the arithmetical average height of surface component (profile) irregularities from the mean line within the measuring length used to describe the vertical dimension of roughness.

Ra=1L∫0Lr(x)dx

Sm is the mean spacing between peaks known as roughness spacing parameter that used to describe the horizontal dimension of roughness.

Sm=1N∑n=1NSn

R_max_ is the maximum roughness depth or the largest peak-to-valley depth over the sampling length. It was recorded to determine any major surface defect.

The data were analyzed statistically using the SPSS software (Version 11.5, SPSS Inc., Chicago, IL, USA) and graphically plotted by Sigma (Σ) Plot (SigmaPlot 2002 ver. 8, SPSS Inc., Illinois, USA). One-way analysis of variance (ANOVA) followed by Scheffé post-hoc test were used to detect the area of significant differences for surface roughness parameters between the composite series at α=0.05. A regression analysis was used to determine possible correlation between different particle sizes of these series and the vertical surface finish (R_a_) as well as the horizontal (Sm) parameters.

## RESULTS

The mean and standard deviation values (µm) of surface roughness parameters (R_a_, Sm, R_max_) for each composite series are summarized in Table 2. One-way ANOVA was used to delineate the areas of significant differences between the composite series. It revealed highly significant differences between the materials for the R_a_ surface roughness parameter (p<0.05). Multiple comparisons Scheffé pos-hoc test showed high significant differences between S-250 as well as I-450 and the following irregular-shape filler composite series; monomodal (I-1500), bimodal (I-450/1000), trimodal (I-450/ 700/1000). Also there were significant differences between S-100 and all the spherical as well as the irregular series except with I-450 and S-250. Moreover, there were no significant differences between all composite series investigated for R_max_ and Sm roughness parameters (p>0.05).

**Table 2:** Mean and standard deviation (sd) of surface roughness parameters (pm) for the experimental composite series

**Roughness parameter**	**Surface roughness mean & sd (µm) of the experimental resin-composite series**										
	**RZD**	**RZD**	**RZD**	**RZD**	**RZD**	**RZD**	**RZD**	**RZD**	**RZD**	**RZD**	**RZD**
	**102**	**107**	**106**	**105**	**114**	**103**	**108**	**109**	**110**	**111**	**112**
Ra	0.079	0.096	0.106	0.106	0.117	0.093	0.105	0.117	0.121	0.124	0.125
	(0.013)^a^	(0.002)^ab^	(0.010)^bc^	(0.011)^bc^	(0.005)^bc^	(0.008)^ab^	(0.009)^bc^	(0.010)^bc^	(0.004)^c^	(0.004)^c^	(0.011)^c^
Sm	267	178	158	212	138	157	176	113	147	142	135
	(51)^b^	(14)^ab^	(17)^ab^	(93)^ab^	(19)^ab^	(81)^ab^	(26)^ab^	(15)^a^	(42)^ab^	(25)^ab^	(40)^ab^
Rmax	0.794	1.096	0.792	1.211	2.379	1.421	0.861	1.374	1.103	0.754	1.259
	(0.178)^a^	(0.751)^a^	(0.073)^a^	(0.541)^a^	(0.334)^a^	(1.049)^a^	(0.131)^a^	(0.393)^a^	(0.411 )^a^	(0.489)^a^	(0.101)^a^

*Superscript letters indicate homogenous subsets (within which *p*>0.05) where comparison has been made with respect to different composite series.

**Figure 2:**
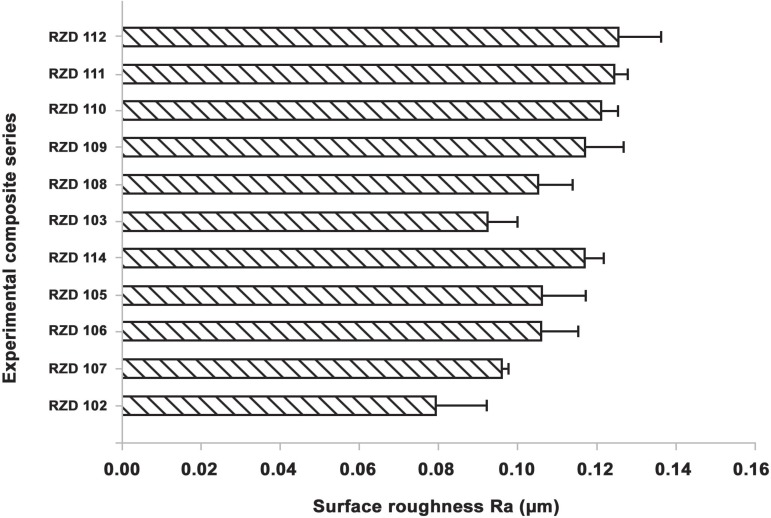
Surface roughness, R_a_ (µm) of the mono-, bi- and multi-modal experimental composite series

**Figure 3:**
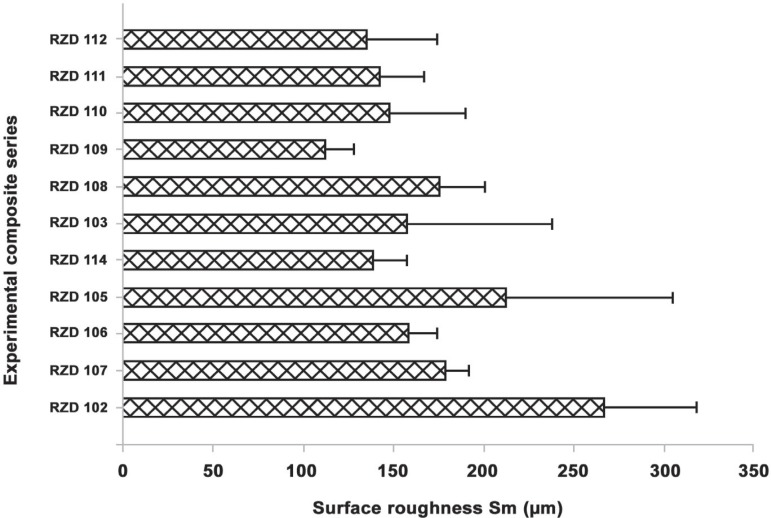
Surface roughness, Sm (µm) of the mono-, bi- and multi-modal experimental composite series

Among the experimental series investigated, the lowest surface roughness (R_a_) was noted in monomodal spherical-shape series; S-100 (0.079±0.013) and S-250 (0.096±0.002). On the other hand, the roughest surface (R_a_) was found in bi- and tri-modal irregular-shape composite series I-450/1000 (0.124±0.004) and I-450/700/ 1000 (0.125±0.011) followed by spherical trimodal series (S-100/250/1000) (Figure 2). Additionally, the latter series expressed the highest R_max_ value (2.379±0.334) whereas S-500 monomodal series showed the lowest value (0.792±0.073). Moreover, I-1000 showed a low Sm value (113±15) compared to S-100 (267±51) (Figure 3). However, the most commonly used parameter to describe roughness is the vertical one (R_a_); and it is compared with the horizontal parameter (Sm).

**Figure 4:**
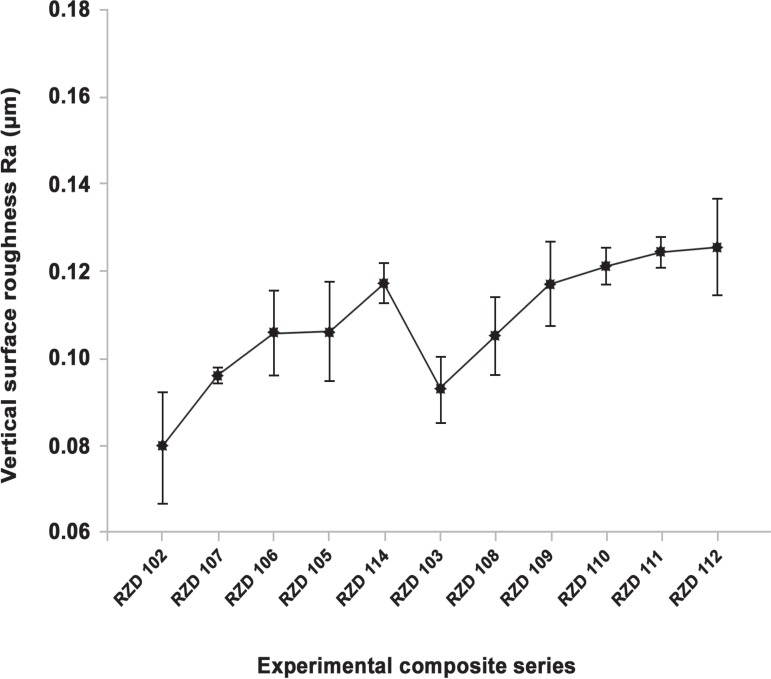
Vertical surface roughness, Ra (µm) regression of the experimental composite series

**Figure 5:**
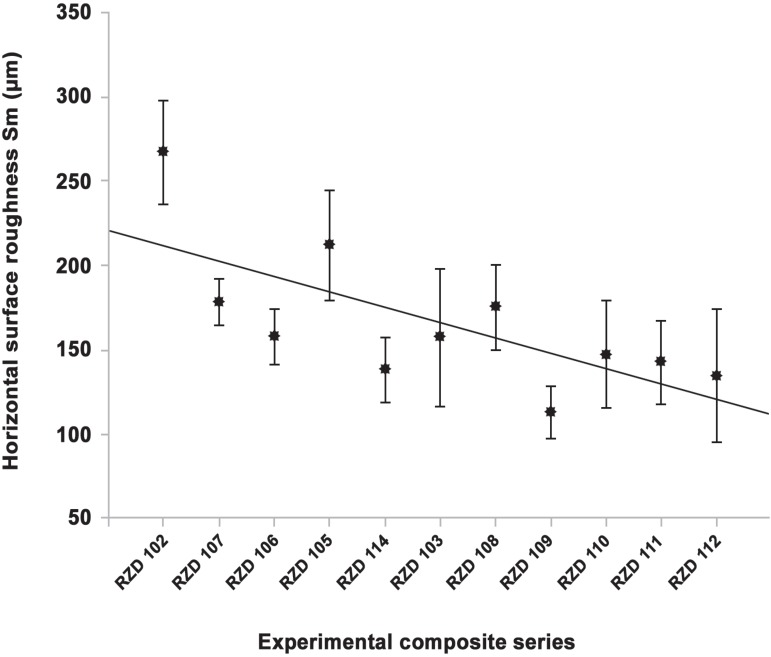
Horizontal surface roughness, Sm (µm) regression of the experimental composite series

The regression analysis showed an increase in R_a_ values with increasing filler particle size yielded a high correlation of both spherical and irregular series (r^2^=0.82), as shown in Figure 4. However, the same analysis demonstrated a non-significant correlation between the increase of filler size and the Sm parameter giving a non-linear regression (r^2^=0.24), as illustrated in Figure 5.

The spherical-shape composite series clearly showed the smoothest surface finish, while the irregular-shape composite series provided the roughest one. In both filler geometries, the surface roughness values gradually increased with increasing the filler size. Moreover, the multimodal series expressed the highest roughness values than the monomodal series especially when they are irregular in shape.

## DISCUSSION

Several methods are currently available to measure the surface texture of any material including contact stylus tracing, scanning electron microscopy, laser reflectivity, non-contact laser metrology and compressed air measuring^15^_._ The most recent method is atomic force microscopy (AFM) and the most common one is the contact stylus tracing^22^. The latter method was used in the current study because it was fast, simple and reliable for comparative assessment of surface roughness property.

In the present study, the resin-rich layer that forms a smooth surface resulted from adaptation of Mylar strip during specimen fabrication was removed by light finishing-polishing procedures. This unpolished surface is usually smoother than the polished one due to the former contain more polymer matrix than the latter. A previous study, however, has shown no significant difference in surface roughness between polished and unpolished surfaces for mainly nanofill resin composites^17^.

Vertical roughness parameters such as R_a_, R_max_, R_t_ and R_z_ are used to describe the surface irregularities by their amplitudes only. The roughness height (R_a_) parameter is merely used by many investigators to estimate the surface quality of resin composite materials. In this study, the spacing parameter (Sm) that measures the horizontal feature of the surface was recorded. Additionally, R_max_ was monitored to determine if any major surface defect on the surface was encountered.

The roughness parameters are dependent on several factors such as filler size, percentage of surface area occupied by filler particles, hardness, degree of conversion of polymer to resin matrix and filler/matrix interaction, as well as stability of silane coupling agent^3,5,8^. Eleven different composite series containing spherical and irregular shape fillers ranged from 100 to 1500 nm and based on mono-, bi- and multimodal (trimodal) filler formulations were studied. The differences in the surface roughness parameters of these composite series might be ascribed to variation in their filler size, geometry and composition.

In the current study, the surface roughness (R_a_) values of the composite series were ranged between 0.079 and 0.125 µm. The monomodal spherical-shape series with a small particle size (S-100) expressed the lowest surface roughness among the materials investigated, while the multimodal irregular-shape series (I-450/700/ 1000) with different particles sizes showed the highest roughness value.

On the scale of filler components, variation in the interparticle spacing, filler distribution, presence of filler agglomeration and clusters, as well as the quality of filler adhesion to the matrix may have an effect on the surface characteristics of these series. Currently, smaller size filler particles can be adhered to resin matrix, thus providing a smoother surface finish^17^. It has been shown that the introduction of finer particles among larger ones will result in reduction of interparticle spacing and the amount of resin matrix, thus maximizing the overall properties of the material^5^. Decreased interparticle spacing caused by reduced filler size may leads to reduction in strain localization around the filler, thus reducing the fatigue failure^9,19^. The concept of multimodal fillers enables the composites to obtain high filler loading and allows a strong integration of small particles into resin matrix that can be eroded by breaking off small individual particles rather than large ones^13,19,20^.

These composite series are dependent on variation in their filler component that differs mainly in size and shape. The low surface roughness of the spherically based composite series could be attributed to that these particles were made from silica, while the irregular particles were ground glass melts. The spherical particles may allow more flow and stress relaxation of the material compared to irregular ones. Theoretically, it was found that spherical particles can be debonded more easily from the matrix than the irregular fillers^19,23^.

In this study, it was noted that the surface roughness values increased with the increase of the filler particle size and also with irregular-shape fillers. This is in agreement with a previous study concluding that a higher surface roughness is associated with larger filler particles^3,14,16^. It was evident that the irregular filler series of the same filler size as I-1000 is rougher than the S-1000. The variation in particle size as in multimodal series may affect the surface roughness of the material through their surface area and interparticle spacing.

As the filler volume fractions were the same in all the series (56.7% vol), the possible explanation for higher surface roughness of multimodal-irregular than multimodal-spherical is that the latter has smaller distance between neighboring particles as compared to the coarser filler particles. Moreover, larger particles especially the irregular ones tended to protrude from the surface, which may result in their high surface roughness. From microstructure perspective, the stress concentration around the irregular fillers may lead to their pull-out from the surface, thus increasing the surface roughness of these series. Another possible explanation could be related to the formation of filler clumping-clusters in multimodal series that may contribute to their high roughness values in comparison to monomodal series. This can be in accord with a previous finding suggested that nanofilled composite expressed high wear resistance and had lower roughness level compared to nanohybrid composites^2,17,19^.

On the other hand, the highest roughness values presented by the horizontal parameter (Sm) were recorded for the monomodal spherical series (S-100), which expressed the lowest vertical roughness value (R_a_). The smaller size fillers provided less vertical dimension; however, they can result in filler agglomeration which may responsible for increasing the horizontal dimension of the roughness profile. Another possible explanation can be related to the ease in flattening of the spherical fillers during lightfinishing that was done in the process of specimen preparation.

However, the lowest Sm value was demonstrated by monomodal irregular type of series (I-1500), which can be explained by presence of surface projection irregularities that may minimize the average spacing between peaks. Moreover, the trimodal irregular series showed an intermediate Sm value between the upper and lower range that demonstrated in the current study. This can be attributed to the presence of multi-filler sizes that minimize somewhat the inter-particle spaces. Therefore, the variation between these two surface roughness parameters (vertical and horizontal) for both spherical and irregular based composite series may be related to the function of their microstructure.

Further investigation is needed to study the same series with a more sensitive device such as a 3-D atomic force microscopy (AFM), which may give a detailed illustration of the surface roughness especially for these composites.

## CONCLUSIONS

Within the limitations of this in vitro study, the following conclusions can be made:

1-The first null hypothesis is rejected since a significant variation in the surface roughness values of the experimental composite series was found.

2-Filler particle size plays an important role in the surface characteristics of the experimental composite series. The vertical roughness value (R_a_) increases as the filler particle size is increased, thus rejecting the second null hypothesis (regarding vertical roughness).

3-The horizontal surface parameter (Sm) of the series is insignificantly correlated with the increase in the filler particle size, therefore, the second null hypothesis could not be rejected (regarding horizontal roughness).

4-Monomodal series with spherical and small size fillers showed the smoothest surface, while multimodal series with irregular and variant filler sizes exhibited the roughest surface parameters.
